# Oral exposure of low-dose bisphenol A promotes proliferation of dorsolateral prostate and induces epithelial–mesenchymal transition in aged rats

**DOI:** 10.1038/s41598-017-18869-8

**Published:** 2018-01-11

**Authors:** Dong-Yan Huang, Cheng-Cheng Zheng, Qi Pan, Shuang-Shuang Wu, Xin Su, Lei Li, Jian-Hui Wu, Zu-Yue Sun

**Affiliations:** 1 National Evaluation Centre for the Toxicology of Fertility Regulating Drugs, Shanghai Institute of Planned Parenthood Research, Shanghai, 200032 China; 2 Reproductive and developmental research institute of Fudan university, Shanghai Institute of Planned Parenthood Research, Shanghai, 200032 China; 3 Fudan University, Shanghai, 200433 China

## Abstract

Bisphenol A (BPA) is a well-known endocrine disruptor compound reported to have prostate toxicity. This study aimed to assess the effect of BPA on the proliferation of dorsolateral prostate (DLP) and the expression of epithelial–mesenchymal transition (EMT)-related genes in aged rats. Male aged SD rats were treated with BPA (10.0, 30.0, and 90.0 µg/kg i.g., daily) or vehicle (i.g., daily) for 3 months. Treatment with BPA resulted in increased the expression of PCNA, DLP weight and DLP epithelial height compared with the control group (*P* < 0.01); such effects were more obvious at higher BPA doses. 90 µg/kg BPA significantly increased the estrogen to androgen ratio (*P* < 0.05). The EMT chip showed the BPA induced upregulation of vimentin, Snail, Twist1, and transforming growth factor beta 1, as well as the downregulation of E-cadherin in the DLP. Immunohistochemical data showed that the expression of vimentin, estrogen receptor subtypes, and androgen receptor increased and the expression of E-cadherin decreased in 30 and 90 µg/kg BPA groups. It was concluded that environmental exposure to low doses of BPA might promote the proliferation of DLP in aged rats by increasing the estrogen to androgen ratio and inducing EMT.

## Introduction

Benign prostatic hyperplasia (BPH) is a common disease in elderly men. The incidence of BPH has shown a gradual upward trend in recent years with the aging of the population and increase in the number of elderly males^1,2^. The prostate is an androgen-dependent organ, and androgen plays an important role in BPH occurrence^3^. Moreover, the effect of estrogen on prostate should not be underestimated. The literature reported that 50 µg/kg estrogen stimulated the development of prostate hyperplasia in Sprague–Dawley (SD) rats^4^, suggesting that low doses of estrogen could promote BPH. However, in a certain range, estrogen can play a synergistic role in promoting BPH^5^. In addition to the endogenous estrogen, scholars focus on environmental estrogens, namely environmental endocrine disruptors (EDCs).

Generally, EDCs disrupt the original endocrine system in the form of hormones after aggregating in organisms^6^. Bisphenol A (BPA), a common xenoestrogen, widely exists in fillers, polymer materials, cosmetics, and plasticizers. A growing evidence suggests that BPA has the potential to cause adverse outcomes in the reproductive system, including prostate. It was found that 235 mg/kg BPA decreased dorsolateral prostate (DLP) weight^7^ and 3 mg/kg BPA increased ventral prostate weight^8^, suggesting that the impact of BPA on the prostate was lobe selective, and the biological effects changed with dose. The oral administration of low-dose BPA was found to promote the proliferation of ventral prostate and upregulate the expression of prostaglandin D2 synthase in adult rats^9^. The biological endocrine system changes with age. BPH is an age-related disease. However, a direct relationship between low-dose BPA and prostate in aged rats has not been demonstrated yet, let alone the DLP.

The exact pathogenesis of BPH is controversial due to various influencing factors. Epithelial–mesenchymal transition (EMT) is an important mechanism that allows the polarized and immotile epithelial cells to convert into motile mesenchymal cells^10^. A previous study indicated that BPH was not the proliferation of stromal cells, but the accumulation of mesenchymal-like cells derived from the prostatic epithelium^11^. EMT is extremely common in tumors. Nanomolar concentrations of BPA can promote the metastasis of colon cancer cells through EMT. Since BPA can induce EMT and EMT can induce BPH, it is speculated that BPA can promote BPH by EMT.

The present study aimed to assess the effect of BPA on the proliferation of DLP and its impact on gene expression related to EMT in aged rats.

## Results

### Body and DLP weight

**Figure 1:**
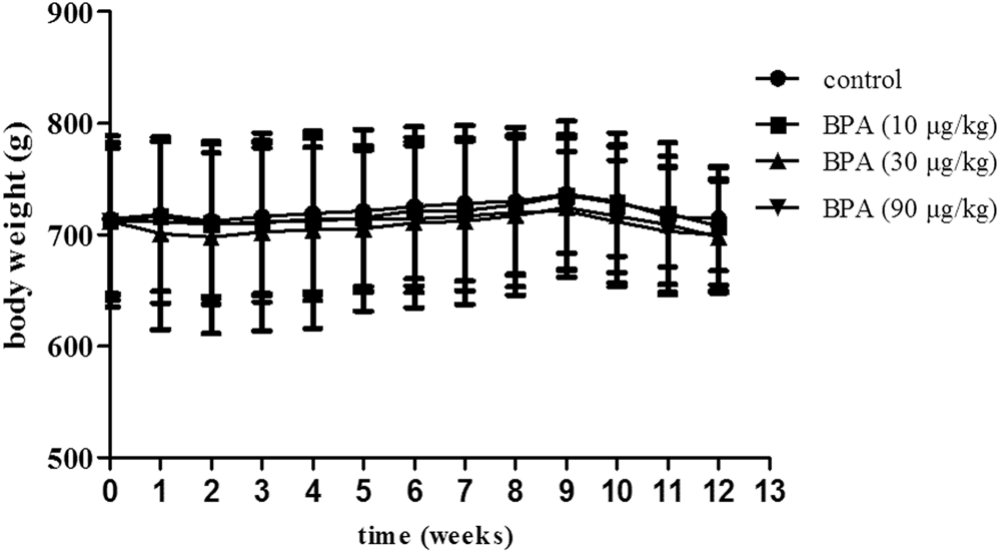
Effect on body weight. After aged rats were treated with 10–90 μg/kg BPA for 3 months, BPA did not have a significant impact on body weight. BPA: bisphenol A.

During the 3-month administration of BPA, animal body weight increased slowly with time, reached the maximum in the ninth week, and then decreased slightly (Fig. 1). After treatment, 10–90 μg/(kgday) BPA did not have a significant impact on body weight; however, BPA increased the DLP weight in a dose-dependent manner. BPA had the trend to increase the volume and relative weight of DLP in a dose-dependent manner, and 90 μg/(kg.day) BPA showed a significant difference (*P* < 0.05, Table 1). However, the volume and relative weight of VP did not change significantly.

**Table 1:** Effects of BPA on body weight and dorsolateral prostate in aged rats treated daily for 3 months (mean ± SD; n = 8).

Group	Treatment (μg/kg)	Body weight (g)	Dorsolateral prostate		Ventral prostate		Volume (ml)
			Weight (g)	Relative weight^a^	Weight (g)	Relative weight^a^	
Control	0	714.6 ± 46.9	0.49 ± 0.08	0.69 ± 0.12	0.65 ± 0.14	0.91 ± 0.16	1.15 ± 0.18
BPA	10	707.6 ± 52.6	0.54 ± 0.08	0.77 ± 0.12	0.75 ± 0.08	1.08 ± 0.15	1.39 ± 0.23
	30	698.1 ± 50.1	0.50 ± 0.05	0.72 ± 0.10	0.72 ± 0.12	1.02 ± 0.15	1.24 ± 0.11
	90	700.1 ± 50.2	0.61 ± 0.13	0.86 ± 0.16^*^	0.73 ± 0.12	1.04 ± 0.16	1.37 ± 0.21

^a^Relative weight: (organ weight/terminal body weight) × 1000.

^*^ *P* < 0.05: compared with the vehicle control.

### Histology

**Figure 2:**
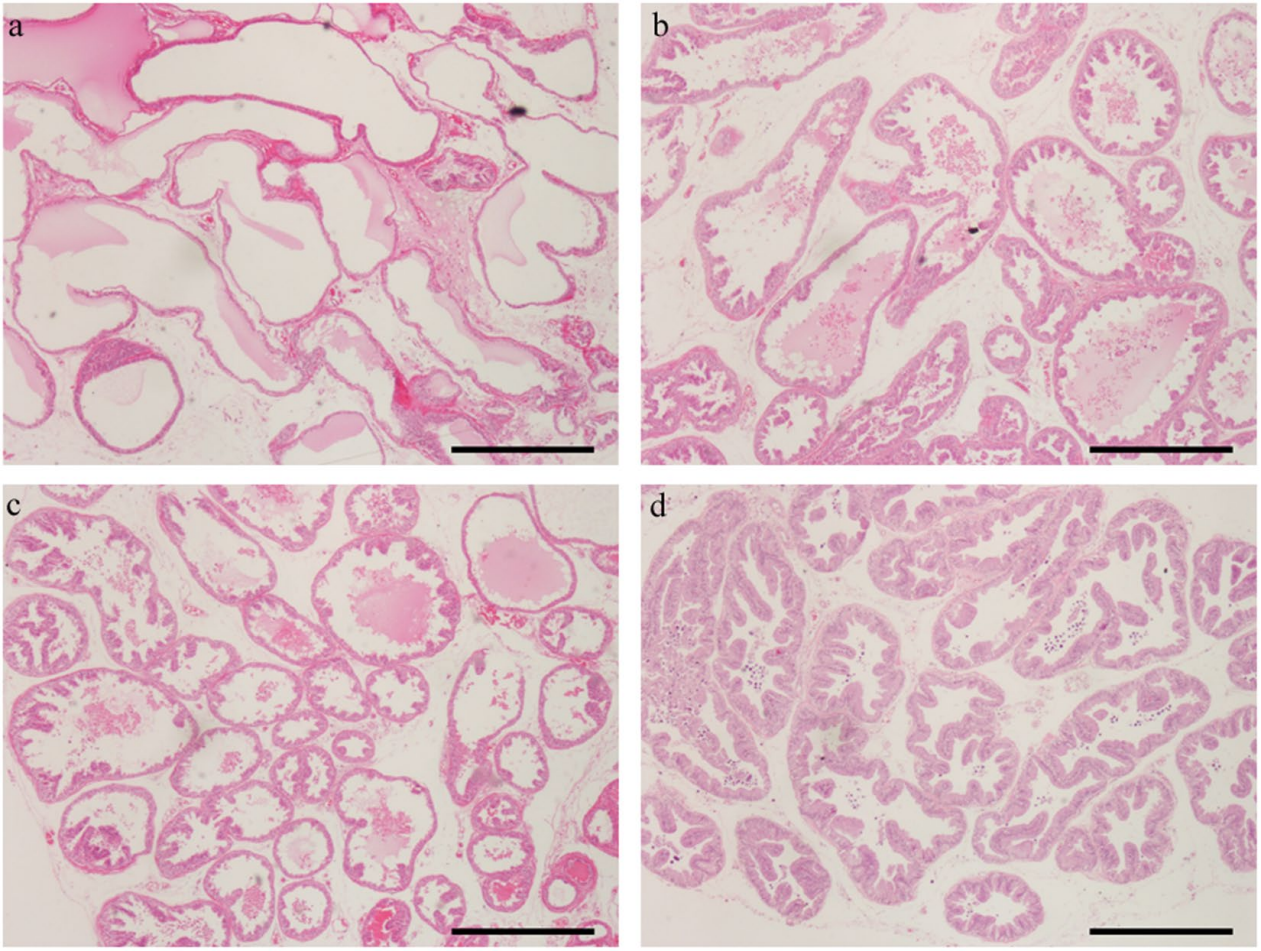
Histological analysis of dorsolateral prostate in male aged rats treated with BPA for 3 months. The glandular cavity was slightly enlarged and increased in BPA-treated groups. (**a**–**d**) Representative sections of comparable regions were shown for vehicle control rats (**a**), and animals exposed to BPA (10 μg/kg/day) (**b**), BPA (30 μg/kg/day) (**c**), and BPA (90 μg/kg/day), (**d**) (scale bar: 50 μm, x40).

**Figure 3:**
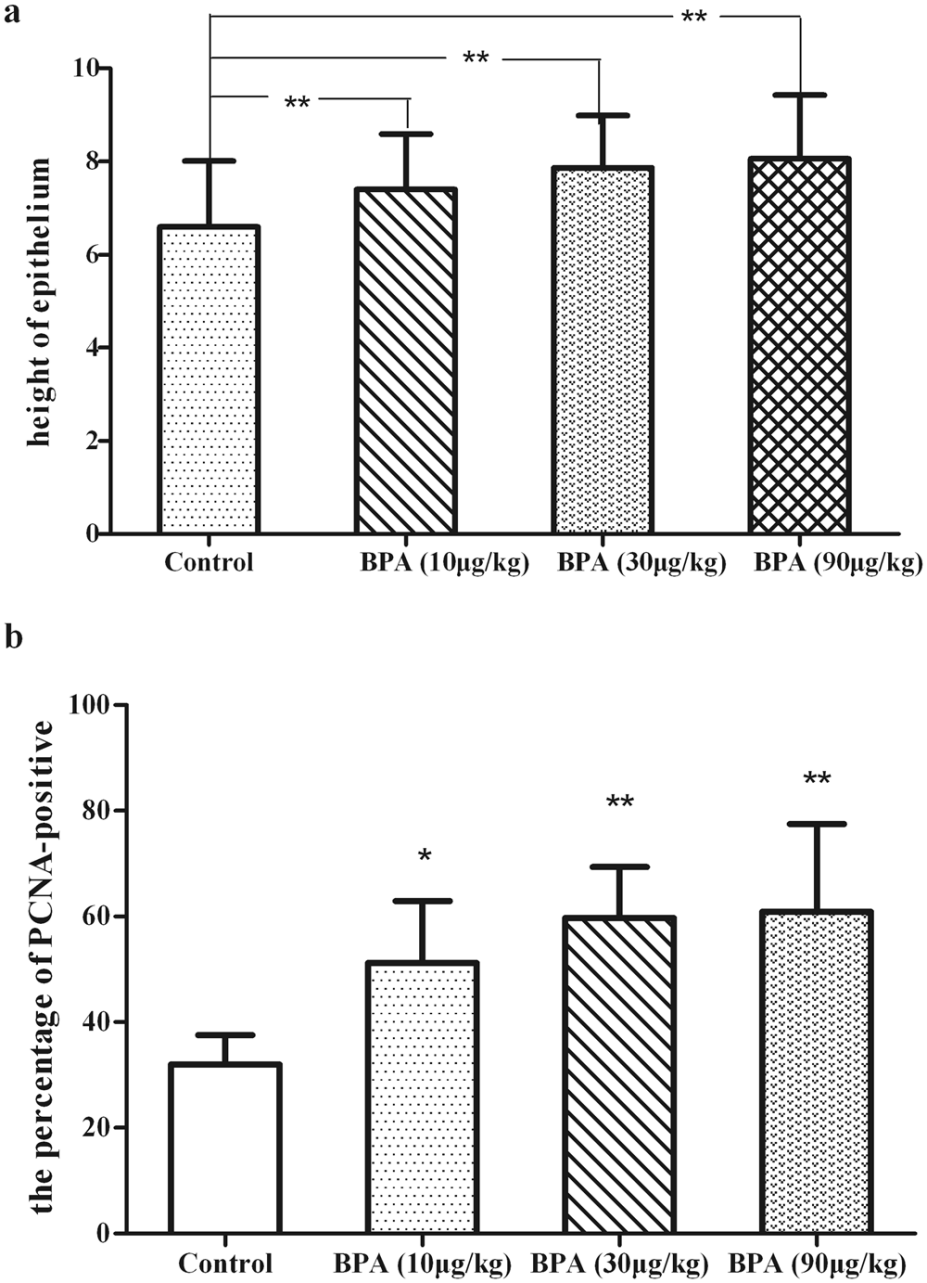
(**a**) Effect on height of dorsolateral prostatic epithelium. After aged rats were treated with 10–90 μg/kg BPA for 3 months, BPA significantly increased the height of DLP epithelium in a dose-dependent way, *P < 0.01, compared with the vehicle controls. BPA: bisphenol A. (**b**) The expression of the PCNA in DLP. The expression of PCNA was increased obviously in BPA-treated groups. *P < 0.05: compared with the vehicle controls; *P < 0.01, compared with the vehicle controls. BPA: bisphenol A.

H&E staining showed glandular prostatic hyperplasia with an increase in the size of alveoli and degree of papillary infolding; the glandular cavity was slightly enlarged and increased in BPA-treated groups compared with the control group (Fig. 2). Moreover, BPA significantly increased the height of DLP epithelium (*P* < 0.01, Fig. 3a); 90 μg/(kg.day) BPA had the most obvious manifestation. However, no significant difference was found among BPA-treated groups. After 3-month treatment, low-dose BPA showed a growth-promoting effect on the DLP.

### Evaluation of the PCNA expression in the DLP

**Figure 4:**
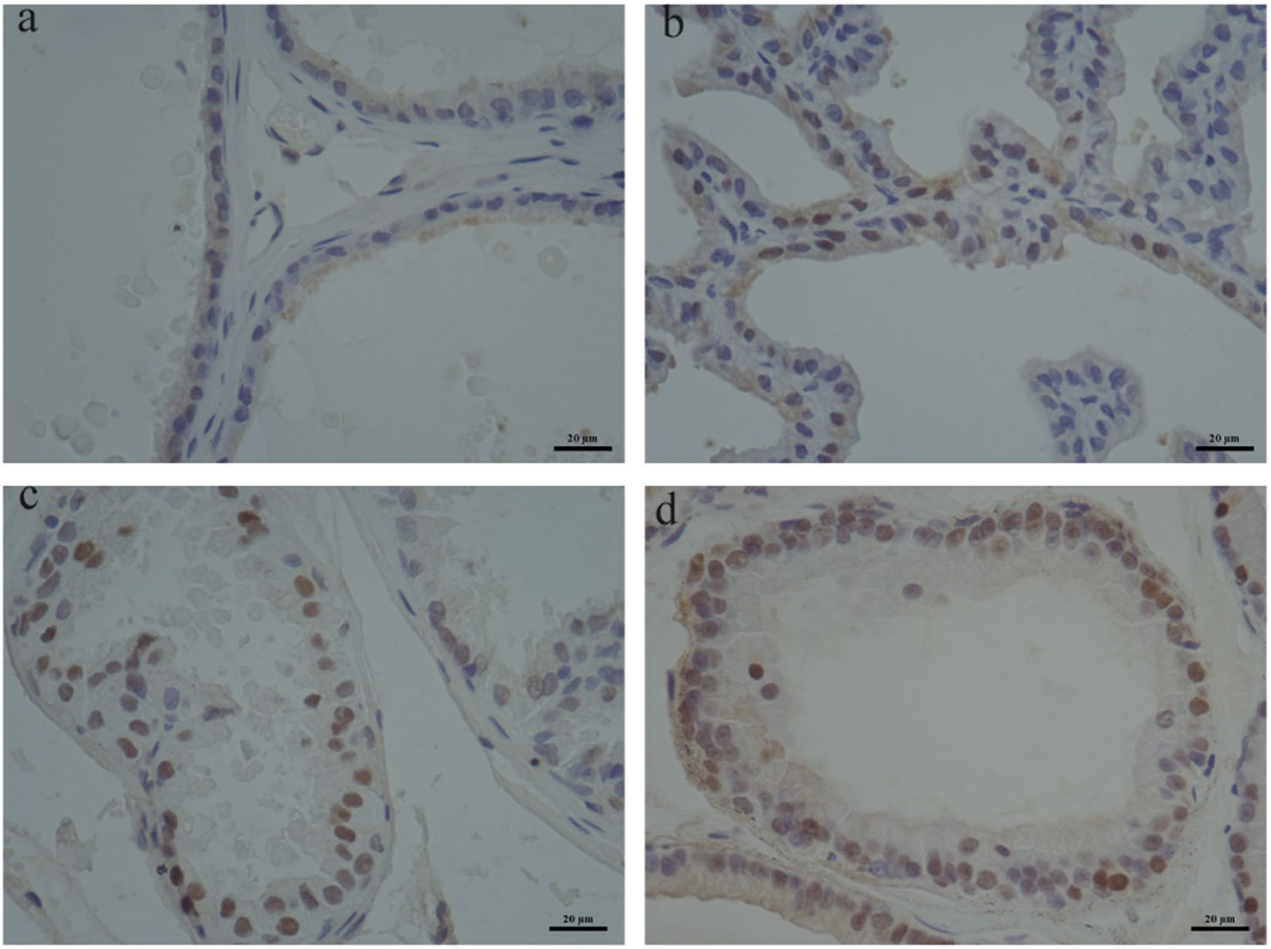
Immunohistochemical analysis of PCNA in DLP. Representative sections of comparable regions were shown for vehicle control rats (**a**), and animals exposed to BPA (10 μg/kg/day) (**b**), BPA (30 μg/kg/day) (**c**), and BPA (90 μg/kg/day), (**d**) (scale bar: 20 μm, ×400).

To better evaluate BPA effect on DLP proliferation, immunohistochemistry for proliferation markers (PCNA) was performed. In the epithelium, positive staining for PCNA in BPA-treated groups was obviously compared with the control group (Fig. 4), the percentage of PCNA-positive epithelial cells in BPA-treated groups was 51%, 60% and 61%, respectively (Fig. 3b).

### Serum hormone levels (estradiol, T, and insulin)

All groups treated with BPA had a higher E_2_ level compared with the control group, especially for 90 μg/(kgday) BPA (*P* < 0.05). A dose of 10 μg/(kgday) BPA increased the serum T level slightly, followed by a trend of decrease with increasing doses. Beyond that, BPA increased the estrogen to androgen ratio, and the increase was more pronounced at a high-dose BPA. The trend was similar for the insulin level between groups treated with BPA and vehicle. These data are shown in Fig. 4.

### EMT gene expression profile of DLP shown by a microarray analysis

**Figure 5:**
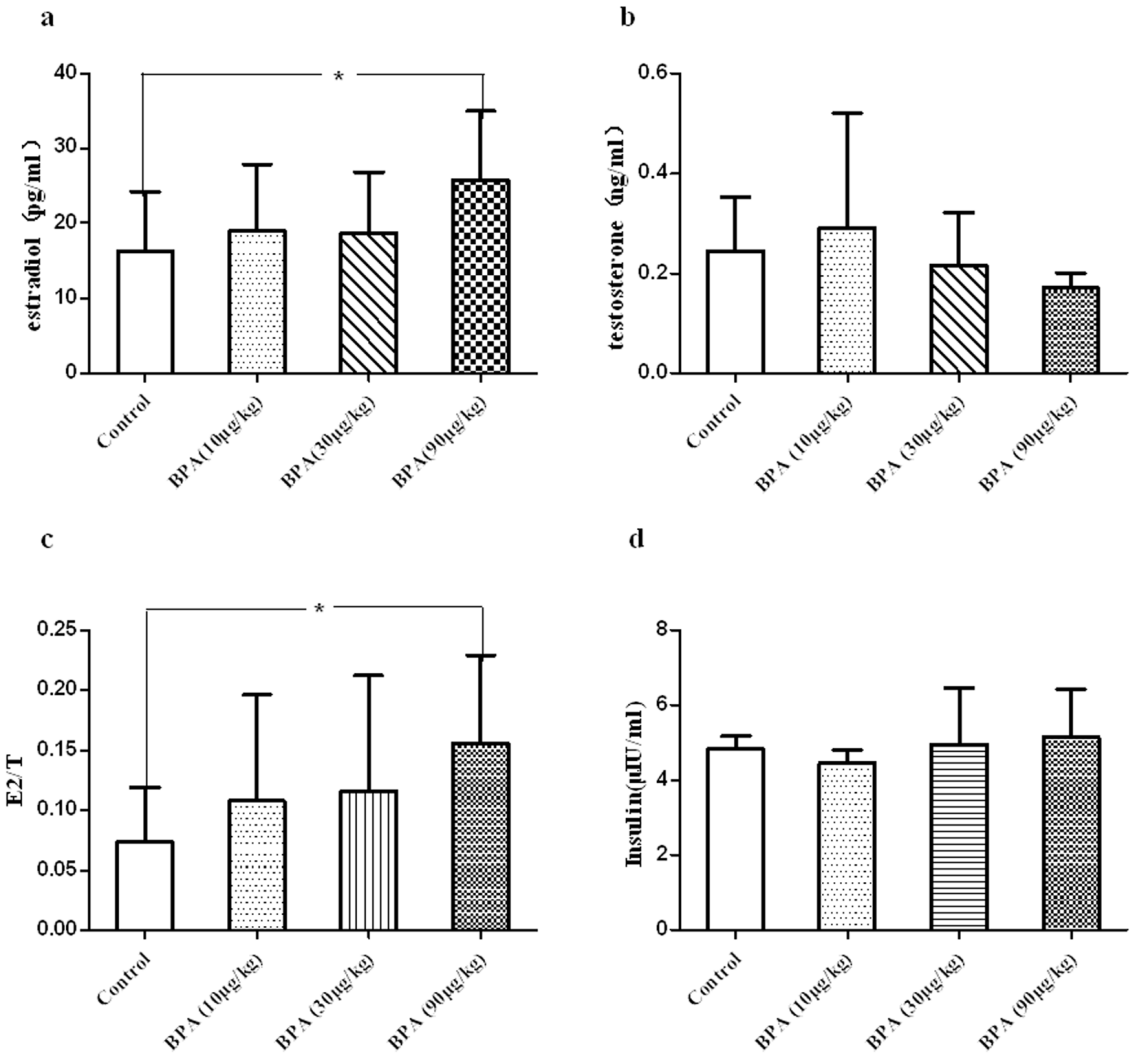
Effects of BPA on E_2_, T, and Insulin serum levels in aged male rats. After aged rats were treated with 10–90 μg/kg BPA for 3 months, 90 μg/kg BPA significantly increased the E_2_ level and the estrogen to androgen ratio; BPA had the trend of decreasing the T level and increasing the insulin level. p < 0.05, compared with the vehicle controls. BPA: bisphenol A.

Gene expression microarray related to EMT was assayed to understand whether a cause-effect relationship existed between BPA and EMT pathway. Quantitative polymerase chain reaction quality results showed that the purity of the samples could meet the experimental requirements. According to scatter plots and data clustering analysis, the gene expression profile was different between the BPA and control groups (Fig. 5). A total of 89 EMT genes were analyzed and 26 genes upregulated at a cutoff of 2 significantly. Statistical analyses revealed the loss of epithelial marker, E-cadherin, and gain of the mesenchymal marker, vimentin, indicating BPA-induced EMT in DLP tissues. Simultaneously, the expression of Snails, Twist, transforming growth factor beta 1 (TGF-β1), and ERα was upregulated (Table 2).

**Table 2:** Effect of BPA on the expression of EMTgenes in DLP of aged rats (n = 4).

Gene symbol	Fold change	Gene bank	Gene description
Cdh1	−1.06	NM_031334	Cadherin 1
Cdh2	2.17	NM_031333	Cadherin 2
Esr1	2.02	NM_012689	Estrogen receptor 1
Fgfr2	2.09	NM_001109892	Fibroblast growth factor receptor 2
Foxc2	2.17	NM_001101680	Forkhead box C2
Fzd7	2.17	XM_006226838	Frizzled family receptor 7
Mmp9	2.17	NM_031055	Matrix metallopeptidase 9
Nodal	2.17	NM_001106394	Nodal homolog (mouse)
Notch1	2.14	NM_001105721	Notch homolog 1, translocation-associated (Drosophila)
Pdgfb	2.17	NM_031525	Platelet derived growth factor receptor, beta polypeptide
Snail1	2.17	NM_053805	Snail homolog 1 (Drosophila)
Snail3	2.17	NM_001107439	Snail homolog 3 (Drosophila)
Tgfb1	2.22	NM_021578	Transforming growth factor, beta 1
Twist1	1.99	NM_053530	Twist homolog 1 (Drosophila)
Vim	2.16	NM_031140	Vimentin
Vcan	−2.57	NM_001170558	Versican
Wnt11	2.17	NM_080401	Wingless-type MMTV integration site family, member 1
Wnt5b	2.17	NM_001100489	Wingless-type MMTV integration site family, member 5

### Evaluation of E-cadherin, vimentin, ERα, and AR expression in the DLP

**Figure 6:**
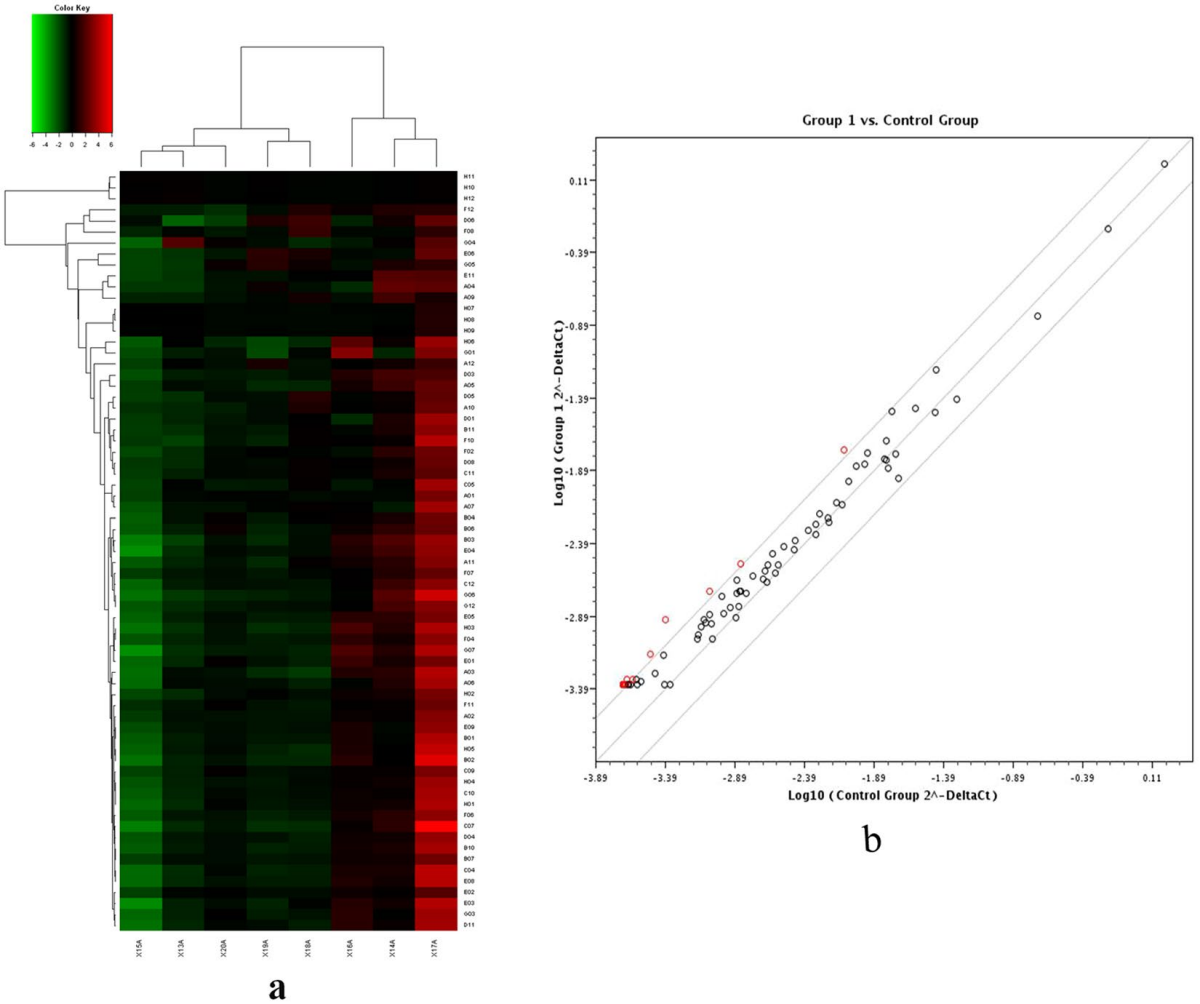
Clustering analysis and scatter plot of microarray data. (**a**) Clustering analysis; (**b**) scatter plot; Group1: dorsolateral prostate of the BPA group; Group control: dorsolateral prostate of the control group. BPA: bisphenol A.

The expression of AR, ERα, E-cadherin, and vimentin was immunohistochemically analyzed to verify further the BPA-induced signaling pathway related to EMT observed in a microarray analysis. The results showed that E-cadherin and vimentin were mainly expressed in epithelial and stromal cells, respectively. The expression of E-cadherin decreased and the expression of vimentin increased gradually with the increase in dose compared with the control group (*P* < 0.05, *P* < 0.01). ERα was mainly expressed in the nucleus. AR was expressed in both the nucleus and the cytoplasm, but the expression was less in the latter. As the dose increased, BPA upregulated the expression of both ERα and AR incrementally (*P* < 0.05, *P* < 0.01) (Table 3 and Fig. 6).

**Table 3:** The expression of E-cadherin, Vimtein, AR and ERα in DLP (mean ± SD; n = 5).

Group	Treatment (μg/kg)	OD			
		E-cadherin	Vimtein	ERα	AR
Control	0	0.205 ± 0.014	0.183 ± 0.017	0.191 ± 0.006	0.196 ± 0.025
BPA	10	0.190 ± 0.015	0.210 ± 0.013**	0.209 ± 0.005	0.210 ± 0.013
	30	0.185 ± 0.016*	0.199 ± 0.008*	0.232 ± 0.008**	0.230 ± 0.011*
	90	0.172 ± 0.013**	0.232 ± 0.013**	0.253 ± 0.029**	0.249 ± 0.014**

BPA: bisphenol A; AR: androgen receptor; ERα:estrogen receptor-α.

*P < 0.05: compared with the vehicle controls; **P < 0.01: compared with the vehicle controls.

## Discussion

**Figure 7:**
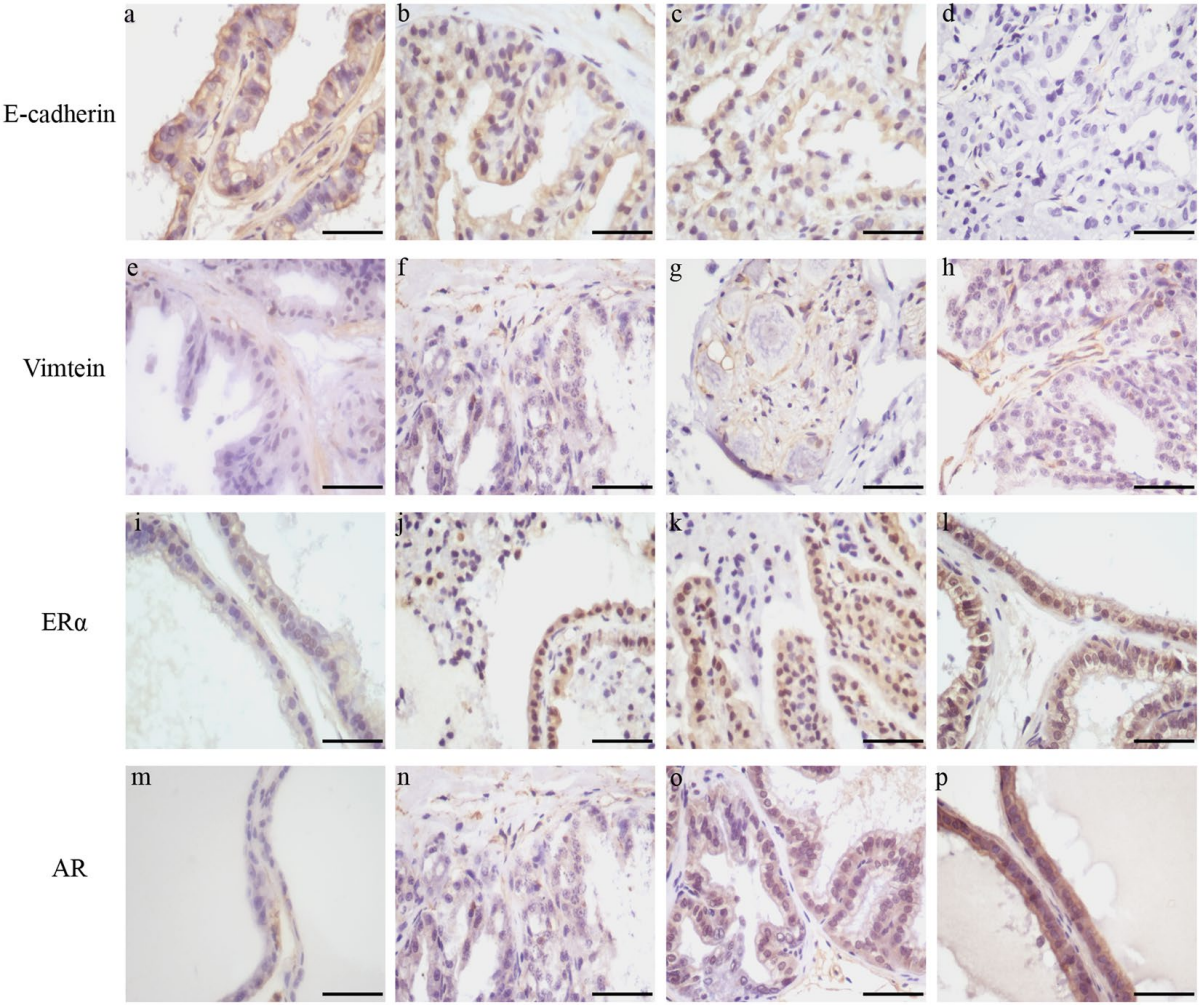
Immunohistochemical analysis of dorsolateral prostate E-cadherin, Vimtein, ERα and AR expression in aged rats. The expression of vimentin, ERα and AR increased, and the expression of E-cadherin decreased in BPA-treated groups. (**a**–**p**) Representative sections of comparable regions are shown for vehicle control rats (**a**,**e**,**i**,**m**), and animals exposed to BPA (10 μg/kg/day) (**b**,**f**,**j**,**n**), BPA (30 μg /kg/day) (**c**,**g**,**k**,**o**), and BPA (90 μg/kg/day) (**d**,**h**,**l**,**p**) (scale bar: 50 μm, ×400). BPA: bisphenol A; AR: androgen receptor; ERα:estrogen receptor-α.

The United States Environmental Protection Agency considers 50 μg/(kg.day) BPA as a relatively safe dose^12^. In this study, less than 50 μg/(kgday) BPA did have the trend of promoting prostate hyperplasia in elderly rats, which was similar to low-dose effects of BPA on a rat model of BPH^4^. Studies have suggested that the injection of 10 μg/(kg.day) BPA in SD rats was equivalent to the BPA detected in serum on environmental exposure^13^. BPA is taken directly into the blood circulation by injection; however, BPA passes through the first-pass effect in oral administration. Therefore, the actual BPA dose given by injection was much higher than that given through the mouth^14,15^. This study showed that 90 μg/(kg.day) BPA could promote the hyperplasia of prostate, which was consistent with the report that the injection of 10 μg/(kg.day) BPA in SD rats increased the incidence of prostate epithelial tumor^13^. Fig. 7.

The proliferation in the DLP was more obvious than that in the VP in transgenic mice because the DLP in mice the mouse was homologous with the peripheral zone in human prostate and BPH originated in the transitional and peripheral zones in humans^16^. After 3-month administration, the DLP was more sensitive to low-dose BPA compared with the VP, especially in the group at 90 μg/kg dose, which also suggested that the DLP in rats might be homologous with the peripheral or transitional zone in human prostate.

Proliferation marker, PCNA, is a proliferating cell nuclear antigen, which is related to DNA repair and cell proliferation. The increase of PCNA expression indicated that the cells were in the proliferation state. In aged rats, serum E2 increased with decreasing levels of serum T, and the E2/T ratio increased with the increase in the BPA dose. Low-dose E_2_ could promote prostate proliferation through the direct stimulation of prostate stromal cells or the regulation of epithelial cells^17^. BPA is considered to have weak estrogenic activity, approximately 1000–10,000 times less than that of E_2_^18^, and it has the same effects on prostate as E_2_. Testosterone (T) can be converted into estrogen by aromatase. Aromatase mRNA expression was found to increase on exposure to BPA, suggesting that BPA decreased T levels on the way of transformation^19^. The study found that 10 μg/kg BPA did not reduce the T level, probably because the exposure level was too low to start the anti-androgenic activity. Estrogen and androgen balance plays an important role in maintaining physiological characteristics and reproductive functions in males. With the increasing age, the androgen level decreases and the estrogen level increases correspondingly. The imbalance of the estrogen/androgen ratio is also considered as one mechanism of BPH^20^. The E_2_/T ratio promotes synergistically prostate hyperplasia between a certain range.

Insulin and insulin-like growth factors promote the proliferation of prostate epithelial cells. It was reported that BPA could combine with ERα or G protein–coupled estrogen receptor (GPER) to increase insulin content and secretion after exposure to low-dose BPA *in vitro* ^21,22^; 10 nM BPA was reported to promote the synthesis of insulin by simulating the effect of estrogen on ERβ and inhibiting the K_ATP_^23^. In this study, the insulin level just tended to increase in the relatively high-dose BPA group, which was not consistent with the results of *in vitro* studies, suggesting the complexity of BPA *in vivo*. The insulin content of adult mice increased after 1-week administration of low-dose BPA. However, the content decreased after continuous administration for 5 weeks. This might be the result of BPA stimulating insulin synthesis by acting on estrogen receptors and inhibiting insulin by downregulating glucose transporter-2 (Glut-2) at the same time, but the promoting effect was the main advantage in a short time^24^.

E-cadherin is the marker protein of epithelial cells mainly distributed in the cytoplasm. E-cadherin belongs to the calcium family. It is a Ca^2+^-dependent transmembrane protein that plays an important role in cell attachment. Vimentin, one of the intermediate filaments, is the marker protein of mesenchymal cells mainly distributed in interstitial cells, participating in the composition of cytoskeleton. This study found that 10 μg/(kgday) BPA significantly upregulated the expression of vimentin and tended to downregulate the expression of E-cadherin. Also, the upregulation of some regulatory factors in charge with EMT genes was visible, including Snail, Twist, Wnt, transforming growth factor beta 1 (TGF-β1), ERα, and so on, most of which have been reported to be related to the formation and metastasis of tumors.

Snail belongs to the superfamily of zinc finger transcription factor, and E-box is the E-cadherin proximal promoter. The combination of Snail and E-box inhibits the expression of E-cadherin to start EMT. Snail can also inhibit the transcription of epithelial cell marker cytokeratin-8 (Krt-8) directly^25,26^. Moreover, 10^−5^ M BPA regulated the stability of Snail by the protein kinase B/glycogen synthase kinase-3β (Akt/GSK-3β) signaling pathway and significantly upregulated the expression of Twist, inducing the occurrence of EMT in colorectal cancer cells^27^. TGF-β1 plays a vital role in EMT, and it can directly activate Smad3, thus stimulating the expression of Snail. TGF-β1 can also combine with the TGF-β1 receptor, evoking the phosphorylation of Smad2 and Smad3 and mixing with Smad4 to form a complex. The complex enters the nucleus to activate the EMT gene^28,29^. In addition to the aforementioned genes, the expression of ERα was also upregulated in the BPA group compared with the control group. The expression of snail and vimentin increased three times, while E-cadherin expression was almost halved after 48-h exposure of BPA in ovarian cancer cells. However, Snail and vimentin were rarely expressed, and E-cadherin expression was not significantly different between the control and BPA groups after administering ER antagonists^30^. BPA could increase the Snail expression and induce the occurrence of EMT through the ER-dependent signaling pathway.

Cconsistent with the results of gene chip, the expression of E-cadherin protein decreased and the expression of vimentin protein increased, which verified the occurrence of EMT at the protein level. Estrogen needs to be combined with the receptor to exert an effect. Estrogen receptor belongs to the nuclear receptor family, mainly including ERα and estrogen receptor beta (ERβ), in which ERα has the role of promoting the proliferation of prostate cells. BPA can compete with E_2_ in binding to ERα due to its similar structure to E_2_, and then combines with specific DNA sequences to promote target genes after entering the nucleus^31^. The combination of BPA and ERα leads to the augmentation of ERα expression, correspondingly promoting proliferation. The prostate is an androgen-dependent organ, and AR plays a pivotal role in regulating function, growth, and differentiation of the prostate gland. In this study, the expression of AR gradually increased with the increase in the BPA dose. AR, as a transcription factor, is activated by the ligand, which can combine with a specific androgen response element to stimulate the transcription, but BPA can interrupt this transcription pathway by binding to AR to exert an anti-androgenic effect^32^. Compared with the anti-androgenic activity, BPA showed a stronger estrogenic activity, with a lower affinity for AR than for ER^30^. AR has also been reported to mediate the EMT process by regulating the expression of zinc finger E-box-binding protein 2 in androgen-dependent cells^33^.

## Conclusion

Taken together, it was concluded that environmentally relevant BPA levels could aggravate BPH in aged rats, and the effect was enhanced with the increase in dose, which is not completely consistent with the effects of BPA in a rat model of BPH in previous studies. Moreover, the DLP was more sensitive to 90 μg/kg BPA. For the DLP, BPA increased the estrogen-to-androgen ratio and upregulated ERα and AR expression, so as to further induce the occurrence of EMT. However, which pathways are involved in EMT needs further confirmation.

## Material and Methods

### Animals and housing

Male Sprague–Dawley (SD) rats (5–7 weeks old, weighing 200–220 g) were purchased from Sino-British SIPPR/BK Laboratory Animal Co., Ltd., Shanghai, China. The animals were housed on sawdust bedding in standard polypropylene cages till the age of 1.5 years. Drinking water and pellet diet (Shanghai Shilin Science & Tech Co., Ltd, China) were available ad libitum in glass bottles. The rooms were maintained at a temperature of 20–26 °C and 40–70% humidity under a 12-h:12-h light/dark cycle. All animal procedures were approved by the Animal Care and Use Committee of Shanghai Institute of Planned Parenthood Research and conformed to the Guide for the Care and Use of Laboratory Animals.

### Reagents

BPA (Lot No. 239658, purity, ≥99.5%) was purchased from Sigma–Aldrich Chemical Company (USA). BPA was solubilized in 0.5% sodium carboxymethyl cellulose (Lot No. 20140520; Sinopharm Chemical Reagent Co., Ltd, Shanghai) solution and stored at room temperature.

### Animal treatment

The rats were randomly divided into four groups (*n* = 8) according to body weights after acclimatization. It has been shown that low doses of BPA may induce the proliferation of ventral prostate (VP) in adult rats^9^. Therefore, 10–90 μg/kg and 3 months were selected as the exposure dose and duration, respectively. The animals were treated with BPA (10, 30, and 90 μg/kg, intragastrically, daily) or vehicle for 3 months, and weighed once a week. All animals were anesthetized with pentobarbital sodium and sacrificed on the day subsequent to last treatment. The DLP were dissected, weighed, and divided into three parts. One part was fixed immediately in 10% formalin, and the other two parts were preserved in frozen liquid nitrogen for the follow-up study.

### Histology

After fixing in formalin for 48 h, DLP tissues were embedded in paraffin, sectioned at 4 μm, and prepared for routine hematoxylin and eosin (H&E) staining. Histological changes were observed under an optical microscope (Nikon Eclipse 50i, Japan), and the height of DLP epithelium was determined using the Nikon NIS-Elements BR 3.1 software (Japan). A total of 20 epithelium samples from each animal and 160 epithelium samples from each group were selected for analysis.

### Hormone level detection

The blood samples were collected and centrifuged for 15 min (3000 rpm, 4 °C) to collect serum. Then, the serum was instantly stored at −80 °C for concentration measurement. Serum estradiol (E_2_), T, and insulin levels were assayed according to the instruction in the corresponding enzyme-linked immunosorbent assay kits (NovaTeinBio, Inc, Cambridge, USA and detected by an enzyme reader (Zenyth 200 st, Austria).

### Evaluation of EMT gene expression by a microarray analysis

Total RNA was extracted from four samples in control and 10.0 μg/kg BPA groups using an RNeasy Microarray Tissue Mini Kit (SABiosciences, Qiagen, Maryland 21703, USA), including the optional on-column DNase digestion step described in the handbook. The concentration and purity of RNA were determined by ultraviolet spectrophotometry and denaturing gel electrophoresis. Then, amplification, array hybridization, washing, and scanning were carried out according to the manufacturer protocol. Data were analyzed by using the ΔΔCT method and microarray data analysis software.

### Evaluation of PCNA, androgen receptor, estrogen receptor subtypes, E-cadherin, and vimentin expression by an immunocytochemical analysis

The expression of androgen receptor (AR), estrogen receptor subtypes (ERα), E-cadherin, and vimentin were detected by the streptavidin–peroxidase method in accordance with the 1:100 (PCNA: 1:50) dilution of the first antibody.

The paraffin-fixed sections were deparaffinized with xylene and rehydrated with gradient ethanol. Then, the sections were immersed in 0.01 M sodium citrate buffer (pH 6.0) and heated to boiling in a microwave for 20 min for retrieval, and endogenous peroxidase was quenched with oxidase blocking solution (Reagent A) for 10 min at room temperature. The sections were incubated in normal nonimmune serum (Reagent B) for 10 min at room temperature to block nonspecific binding. The sections were covered with primary antibodies (ERα was purchased from Proteintech Group, Inc, Rosemont, IL 60018, USA; PCNA was purchased from Santa Cruz Biotechnology, Inc; other antibodies were purchased from Wuhan Boster Bio-engineering Company, Wuhan, China) in wet boxes at 4 °C overnight (PCNA: RT I h), but the first antibody was substituted with phosphate-buffered saline in the negative control group. Corresponding secondary antibodies (Reagent C) and *Streptomyces* antibiotic peroxidase solution (Reagent D) were added successively at room temperature for 10 min, followed by staining with a 3,3′-diaminobenzidine kit. The sections were treated with hematoxylin, dehydrated, washed, mounted, and observed under an optical microscope (Nikon Eclipse 50i, Japan). Finally, the mean optical density values were analyzed using the Image-Pro Plus 6.0 software.

### Statistical analysis

Data were analyzed using SPSS version 17.0 (SPSS, IL, USA) and presented as mean ± standard deviation. Statistical comparisons were performed by one-factor analysis of variance. If statistically significant, the differences between control and treatment groups were tested using the least-squares means test.

### Ethical standards

All animals in this study were treated humanely according to the Guide for the Care and Use of Laboratory Animals of Shanghai Institute of Planned Parenthood Research Animal Care and Use Committee.
